# Cultural congruence and educational equity: how ethnic minority teachers promote minority students’ school adaptation and its underlying psychological mechanisms

**DOI:** 10.3389/fpsyg.2026.1739625

**Published:** 2026-03-02

**Authors:** Zipei Ouyang, Liang Jing, Cheng Li, Jiaohui Tang

**Affiliations:** 1 School of Marxism, Quzhou College of Technology, Quzhou, Zhejiang, China; 2 Dongguan University of Technology, Dongguan, Guangdong, China; 3 Hubei Three Gorges Polytechnic, Yichang, China; 4 Department of Education, Graduate School, Kookmin University, Seoul, Republic of Korea

**Keywords:** educational equity, ethnic identity, school adaptation, sense of belonging, teacher cultural congruence

## Abstract

Grounded in a culturally and psychologically informed perspective on educational equity, this study examines how teachers’ cultural congruence is associated with ethnic minority university students’ adaptation to campus life and explores the psychological processes underlying this relationship. Drawing on attachment theory, social comparison theory, and ethnic socialization theory, we developed a structural equation model in which students’ sense of belonging was specified as the central psychological mediator, while teachers’ and students’ ethnic identities were examined as contextual moderators. Using survey data from 180 undergraduates enrolled in three ethnic and normal universities in western China, structural equation modeling indicated that perceived teacher cultural congruence was strongly and positively associated with students’ school adaptation (total effect *β* = 0.66, *p* < 0.001). Further analyses showed that students’ sense of belonging significantly mediated this association (indirect effect *β* = 0.42, 95% CI [0.32, 0.53]), suggesting that a substantial portion of the association between cultural congruence and school adaptation operates through students’ emotional connection to the school environment. Moderation analyses revealed that teachers’ ethnic identity strengthened the association between cultural congruence and students’ sense of belonging (*β* = 0.19, *p* < 0.01), while students’ ethnic identity amplified the association between belonging and school adaptation (*β* = 0.21, *p* < 0.05), indicating a context-dependent in-group cultural resonance effect. The overall model demonstrated good fit to the data (CFI = 0.958, TLI = 0.943, RMSEA = 0.041), and all latent constructs showed satisfactory reliability and validity. Taken together, the findings suggest that teachers’ cultural congruence supports minority students’ school adaptation primarily by fostering a sense of belonging within everyday teacher–student interactions. These results highlight that educational equity is not achieved solely through institutional arrangements, but is actively constructed through culturally attuned relationships and psychological connection in the classroom context.

## 1 Introduction

### 1.1 Global educational equity and the challenge of cultural congruence in minority students’ school adaptation

Educational equity remains a persistent challenge for education systems worldwide, even amid rapid globalization and the steady expansion of schooling. Although access to education has widened across many countries, deep-seated structural inequalities, cultural biases, and social hierarchies continue to influence how different groups learn and develop. The integrative model of minority children’s development proposed by García Coll et al. (1996) highlights that minority students’ learning and socialization are shaped by interlocking factors—social status, cultural identity, and institutional context. Education, therefore, cannot be regarded as a neutral sphere of growth; it often reflects and reproduces broader social inequities. Within this environment, cultural variation is frequently misread as an “ability gap,” leaving minority students disadvantaged both academically and psychologically. This dynamic is particularly salient in highly standardized higher education systems, such as that of China, where uniform curricula, centralized evaluation mechanisms, and dominant cultural norms coexist with policy-level commitments to ethnic diversity and inclusion. This pattern appears across nations with vastly different economic and social structures. In the United States, for instance, a longitudinal study by Suárez Orozco et al. (2010) on newly arrived immigrant adolescents revealed that cultural mismatch and language barriers contribute to long-term academic decline, limiting classroom participation, self-efficacy, and identification with school. Similar experiences have been observed throughout Europe, where numerous studies describe an institutionalized “cultural disconnection” among immigrant and minority youth struggling to establish a sense of belonging in mainstream education systems.

From a sociohistorical standpoint, Ladson Billings (2006) introduced the concept of “educational debt,” arguing that enduring inequities have systematically disadvantaged minority populations in educational policy and resource allocation. She contended that achievement gaps stem not from individual differences in effort but from structural injustices and the consistent neglect of cultural identity. In the Chinese higher education context, where ethnic minority students are formally incorporated into mainstream universities as well as designated ethnic institutions, similar tensions emerge between institutional inclusion and students’ everyday psychological experiences of recognition and belonging. Subsequent evidence reinforces this claim. A meta-analysis by Berkowitz et al. (2017) documented a strong link between socioeconomic status, school climate, and educational outcomes. Students from low-status or culturally marginalized backgrounds often perceive exclusion and unfairness within the school environment, which erodes motivation and persistence. Subtle yet persistent forms of differential evaluation inside schools quietly reproduce inequality.

These psychological barriers are especially pronounced in multiethnic and multicultural contexts. In a comparative study across several European countries, Baysu et al. (2021) found that teachers’ approaches to diversity education crucially shape students’ social and emotional development. Inclusive and culturally responsive teaching practices enhance minority students’ sense of belonging and engagement, whereas assimilation-oriented approaches tend to weaken both. Within Chinese universities, where professional norms often emphasize neutrality and uniform instructional standards, students’ perceptions of whether teachers understand and respect their cultural backgrounds may play an especially important role in shaping emotional security and social integration. A cross-national review by Gorard et al. (2025) further confirmed that teacher–student ethnic congruence exerts a powerful influence on learning outcomes. When teachers and students share cultural backgrounds, students show higher academic performance, greater emotional stability, and stronger participation in social life—a pattern consistently reported in multicultural societies such as the United States, the United Kingdom, Canada, and Australia. Although the structural organization of Chinese higher education differs from these contexts, the psychological significance of teacher–student cultural alignment remains highly relevant, particularly in settings where minority teachers are underrepresented and cultural understanding must be actively negotiated within everyday classroom interaction.

As education systems continue to reform, discussions of equity have shifted away from simply expanding access toward improving the quality and cultural relevance of learning experiences. Yet, many systems still exhibit a deep institutional tendency toward assimilation, leaving minority and immigrant students struggling to maintain belonging and affirm their identities. Achieving genuine educational equity, therefore, requires more than a redistribution of resources; it calls for sustained cultural understanding, stronger emotional support, and the reconstruction of teacher–student relationships within diverse educational communities. In this sense, examining cultural congruence within Chinese higher education offers an opportunity to understand how educational equity is experienced psychologically in a system that is structurally inclusive yet culturally standardized.

### 1.2 From cultural adaptation to cultural congruence: theoretical evolution and the repositioning of the teacher’s role

Over the past decades, studies on cultural adaptation have gradually moved away from early models that emphasized assimilation and integration. The focus of contemporary scholarship has turned toward understanding how different cultures coexist and interact on more equal terms. Initial research in this area mainly investigated how individuals adjusted their thoughts and behaviors to fit dominant cultural expectations. In his influential work on acculturation, Berry (1980) distinguished four orientations—assimilation, separation, integration, and marginalization—to explain the psychological strategies individuals employ when seeking balance in cross-cultural situations. Although Berry’s model laid an essential foundation for later inquiry, it assumed that adaptation flowed from minority toward majority groups and thereby reinforced mainstream cultural norms as the reference point for successful adjustment. Ward (2001) expanded this framework by viewing adaptation as a multidimensional process that unfolds across psychological, social, and cultural domains. Her work drew attention to the ways in which social networks and institutional contexts shape individual experiences, shifting the discussion from personal adjustment to the interplay between learners and their environments. This broader lens opened new possibilities for examining adaptation within educational systems where relationships and settings are inherently complex.

As the twenty-first century unfolded, scholars increasingly questioned whether one-directional models could capture the realities of multilingual and multiethnic classrooms. Reviewing Indigenous education research, Castagno and Brayboy (2008) introduced the idea of culturally responsive schooling. They argued that teaching should not simply accommodate difference but actively incorporate students’ cultural experiences in order to challenge the colonial assumptions embedded in dominant knowledge structures. Their proposal marked a move from passive adjustment to active cultural engagement. Villegas and Irvine (2010) emphasized that diversifying the teaching workforce is critical to the success of culturally responsive education. Teachers, they noted, are not only conveyors of information but also interpreters of cultural meaning whose identities and awareness directly shape students’ sense of belonging. Building on this perspective, Paris (2012) advanced the concept of culturally sustaining pedagogy, urging educators to maintain and develop minority languages and traditions rather than merely adapt them to mainstream expectations. Ladson Billings (2014) reframed this line of work as “culturally relevant pedagogy 2.0,” calling on teachers to cultivate critical cultural consciousness and to use classroom practice as a means of re-examining power and knowledge relations. Gay (2015) further systematized the principles and methods of culturally responsive teaching. She argued that effective instruction balances cognition, emotion, and values so that students can achieve academic success while preserving cultural integrity and self-respect. At the policy level, Banks (2015) advanced a comprehensive model of multicultural education grounded in democratic participation and civic equality, envisioning schools as spaces where cultural differences strengthen social cohesion rather than deepen division. Extending this approach, Khalifa et al. (2016) introduced the notion of culturally responsive leadership, emphasizing that educational leaders must promote cultural justice and structural transformation within their institutions.

Generally, these developments trace a shift from theories of cultural adaptation to those of cultural congruence—from viewing individuals as adjusting to dominant systems toward exploring how education itself can foster cultural equality and psychological reciprocity. This theoretical movement provides the conceptual grounding for the present study: the congruence between teachers and students is understood not only as an affective dimension of classroom interaction but also as a psychological and institutional condition essential to achieving genuine educational equity.

### 1.3 Theoretical gap and innovative contributions: psychological mechanisms of cultural congruence between minority teachers and students

Although research on culturally responsive education has illuminated how teachers’ cultural awareness relates to students’ academic growth, far less is known about the psychological processes through which minority teachers’ cultural congruence fosters students’ adaptation to school life. Most existing studies concentrate on the statistical relationship between “same-ethnicity teacher effects” and student performance, leaving the underlying mechanisms largely unexplored. Using large-scale datasets, Egalite et al. (2015) showed that students paired with teachers of the same ethnic background achieve higher academic outcomes, underscoring the continuing importance of representation for motivation and achievement. Their work, however, dealt only with academic indicators and offered little insight into emotional or social dynamics. Cherng and Halpin (2016) approached the topic from students’ viewpoints, finding that minority teachers often inspire stronger trust and emotional identification during classroom interaction. They also noted that these patterns depend on school culture and on teachers’ own identity awareness, revealing the contextual character of cultural congruence.

Later studies expanded this discussion. Bristol (2019) introduced the notion of teachers’ cultural added value, arguing that minority educators not only transmit knowledge but also build settings of psychological safety and cultural affirmation—conditions vital for the emotional stability and social participation of marginalized learners. Through a long-term longitudinal design, Gershenson et al. (2022) found that early exposure to same-ethnicity teachers predicted higher educational aspirations and greater occupational confidence in later years, indicating that cultural congruence exerts lasting and cumulative influence on students’ development. Much of this evidence, however, derives from North American contexts, and empirical validation in non-Western settings remains limited. In parallel, scholars have begun to examine the mediating function of cultural belonging in school adaptation. Gray et al. (2018) proposed a tripartite model of belonging that links students’ sense of inclusion to relationships, classroom participation, and institutional support, identifying cultural identity as a moderating element. From the standpoint of social support, Wang and Eccles (2012) showed that close teacher–student relationships and emotional encouragement enhance both behavioral engagement and cognitive participation, providing evidence for an affective route through which cultural congruence may operate. More recently, Lai et al. (2023) examined rural schools in China and demonstrated that teachers’ ethnic socialization practices strengthen students’ cultural identity and psychological well-being. Their study highlighted how teachers’ cultural expression interacts with students’ identity formation and contributed important empirical evidence from a non-Western context.

Despite these advances, research still lacks a coherent explanation of the internal structure and cross-cultural consistency of cultural congruence effects. Previous work has largely verified outcomes without constructing integrated models that link congruence, belonging, and adaptation. The present study addresses this limitation by combining insights from theories of culturally responsive teaching, social identity, and belonging. It develops a conceptual framework in which teacher–student cultural congruence shapes school adaptation through students’ emotional belonging and social identification, moderated by teachers’ ethnic identity awareness. By testing this model empirically, the study aims to clarify the psychological pathways of cultural congruence and to provide a theoretically grounded basis for teacher development and culturally informed educational practice in minority regions.

## 2 Literature review

### 2.1 Teacher cultural congruence and educational equity: from theoretical origins to a value reorientation

Debates on educational equity have evolved beyond issues of resource distribution and structural access to address questions of cultural recognition and psychological belonging. The persistence of inequality often stems from cultural biases and institutional exclusions normalized within everyday teaching. In this environment, teachers operate not only as conveyors of knowledge but as creators of cultural meaning. Sleeter (2012) observed that dominant reform narratives have focused narrowly on standardized testing and achievement, neglecting the power relations that structure classroom culture. She framed culturally responsive pedagogy (CRP) as more than a set of strategies—a social-justice approach that ensures students from varied cultural backgrounds are genuinely acknowledged and respected. This understanding redefines educational equity, shifting attention from equal resources toward cultural acknowledgment and urging schools to affirm, rather than simply accommodate, students’ lived experiences. Through a systematic review of Critical Race Theory (CRT) in educational research, Ledesma and Calderon (2015) demonstrated how culture and power intersect in schooling. They showed that racialized norms within curricula, language, and assessment reinforce the dominance of majority cultures and erode the legitimacy of minority ones. The integration of CRT has encouraged scholars to re-examine how institutional injustices take shape in classrooms and to view teachers’ cultural identities and pedagogical awareness as central to understanding equity. Teachers’ cultural sensitivity and reflexivity have thus come to represent acts of resistance against structural bias. Rather than serving as neutral intermediaries, teachers act as cultural agents who participate in redistributing knowledge and reshaping collective understanding. Drawing on qualitative research in urban schools, Milner (2011) found that educators in multiethnic settings often struggle with limited cultural understanding, leading to weak emotional connection and diminished psychological safety for students.

Genuine cultural congruence, therefore, goes beyond simply adding “cultural content” to instruction; it requires teachers to reflect critically on their own positionality and values in everyday practice. In a systematic review of multicultural teacher education programs, Parkhouse et al. (2019) emphasized that cultural responsiveness is learned rather than innate, developed through sustained professional reflection and engagement. Training confined to theory seldom alters classroom behavior, suggesting that cultural congruence should be recognized as a vital part of teachers’ professional formation. Using narratives of minority teachers, Gist (2018) extended this argument by showing how these educators act as cultural mediators who, through identity modeling and empathy, help students rebuild cultural confidence and psychological safety. In this sense, educational equity is not a compensatory response for marginalized groups but a broader process of cultural empowerment. From a methodological standpoint, Joseph et al. (2024) argued that cultural responsiveness must be evaluated through both subjective perception and institutional structure. Their work in Indigenous schools underscored that cultural congruence is relational and dynamic, emerging through interactions among teachers, students, and institutions rather than existing as a fixed attribute. According to the contexts above, these studies illuminate the cultural foundations of educational equity. Teacher cultural congruence is best understood as a core mechanism—rather than a peripheral method—in realizing social justice and psychological equality. Culturally responsive pedagogy thus challenges the illusion of neutrality in education, extending the notion of equity from structural redistribution toward cultural understanding and emotional connection. Viewed in this way, teacher cultural congruence serves as a process of emotional and social reconstruction that provides a renewed theoretical foundation for explaining the inner dynamics of educational equity.

### 2.2 The educational equity significance and theoretical foundations of teacher cultural congruence

Achieving educational equity requires more than sound policy or institutional reform; it also depends on the degree of cultural congruence that shapes teacher–student relationships in daily classroom life. When teachers and students share ethnic or cultural backgrounds, their interactions often build deeper trust and understanding, which can influence students’ academic success, emotional growth, and behavioral adjustment. Dee (2005) offered the first empirical evidence of what he called the teacher–student ethnic match effect. He found that students taught by teachers of the same ethnic background participated more actively in class and achieved better academic results. This finding redirected debates on educational equity, moving attention away from material and structural resources toward its cultural and psychological dimensions and emphasizing the importance of teachers’ cultural identities in sustaining learning motivation and achievement. Building on this foundation, Wright et al. (2017) conducted a longitudinal study in early-childhood settings and reported that ethnic congruence benefited not only academic performance but also emotional stability and social competence. Their work suggests that teachers’ cultural awareness helps establish safe and affirming environments where socio-emotional learning becomes integral to educational equity. Research on school discipline offers a complementary view. Lindsay and Hart (2017) observed that Black students who had previously been taught by Black teachers were less likely to receive disciplinary sanctions. They argued that this pattern reflected teachers’ culturally informed empathy and contextual understanding of student behavior rather than leniency, reducing both misinterpretation and bias. Using large-scale statistical data, Harbatkin (2021) showed that ethnic alignment has lasting effects on course performance and learning motivation, with especially strong benefits for students from disadvantaged backgrounds. From the perspective of school climate, Bottiani et al. (2017) found that students’ sense of fairness and belonging closely mirrors the degree of teachers’ cultural understanding. Where such understanding is limited, students are more prone to alienation and mistrust; where it is present, shared values help rebuild emotional security and provide psychological support against structural inequality.

Scholars have also begun to examine how teacher cultural congruence shapes education systems more broadly. Reviewing Grow Your Own teacher programs, Gist et al. (2019) noted that a more diverse teaching workforce strengthens students’ experiences of cultural identity and helps create lasting structures of cultural representation in schools—an approach that fosters inclusion and long-term equity. Taken together, these studies show that teacher cultural congruence operates beyond individual classrooms and functions as a key mechanism for realizing educational equity within schools and institutions. Its deeper value lies in rebuilding the emotional and cultural foundations of educational relationships. Cultural congruence reframes equity as a process of ongoing interaction rather than a fixed pattern of distribution, enabling teachers and students to construct shared meaning through mutual respect and recognition. Real progress toward equity, therefore, depends not only on systemic protections but on the daily practice of cultural empathy within classroom life.

### 2.3 The mediating role of cultural belonging: a bridge between emotional connection and social identity

Educational psychologists often describe cultural belonging as the bridge between an individual’s inner experience and the wider social world. It shapes students’ emotional balance and commitment to learning while influencing how cultural identity relates to educational adjustment. Strayhorn (2012) viewed belonging as a vital psychological element of academic success: when students feel accepted, understood, and valued at school, they are more likely to sustain motivation and develop a positive self-concept. Those who feel excluded commonly struggle with academic performance, emotion regulation, and social connection, which makes belonging central to explaining the psychological basis of educational equity. Using a daily-diary method, Gillen O’Neel (2021) showed that belonging varies from day to day rather than remaining constant. She observed that students’ sense of inclusion rises and falls with teacher interactions, peer acceptance, and classroom atmosphere. Among first-generation college students, even small positive encounters can strengthen engagement and identity coherence, whereas negative experiences tend to erode psychological safety. This micro-level evidence clarifies how teachers’ cultural awareness and communication shape students’ everyday feelings of belonging, illustrating the emotional pathway through which cultural congruence operates. In work on ethnicity and cultural identity, Kiang and Fuligni (2009) found that belonging differs across relationships with family, same-ethnic peers, and peers from other groups. Family and co-ethnic networks often provide steady cultural support, while cross-group settings may foster exploration but also create tension. Follow-up research (Kiang and Fuligni, 2010) showed that meaning in life mediates the link between cultural identity and psychological adjustment, implying that belonging is more than emotion—it is an ongoing process of meaning-making that joins personal experience with social identity. For minority students, classrooms that respect cultural difference and encourage genuine identity expression reinforce this sense of meaning, supporting both psychological adjustment and academic involvement.

Rivas-Drake et al. (2014) used a multidimensional model of ethnic identity to demonstrate that belonging mediates the connection between psychological development and academic outcomes. They reported that the belonging aspect of ethnic identity is closely tied to mental health and academic self-efficacy. Strong ethnic belonging nurtures self-esteem and social participation, whereas weak or uncertain identification often brings anxiety and isolation. Likewise, Byrd (2014) showed that cross-group contact and teachers’ ethnic-socialization messages shape students’ motivation: those who perceive fairness and cultural inclusion in class tend to persist longer and regulate their learning more effectively. Viewed collectively, this research portrays cultural belonging as the psychological link between teachers’ cultural congruence and students’ adaptation to school. It weaves emotion, identity, and culture into a living system of connection, suggesting that educational equity relies not only on institutions or resources but on whether learners feel genuinely seen and understood in their classrooms. In minority-education settings, teachers’ cultural congruence builds emotional safety and social identification, activating students’ inner resources of belonging and fostering both academic success and mental well-being.

### 2.4 The moderating role of teachers’ ethnic identity and cultural socialization: a contextual and differential analysis

Cultural belonging helps explain the inner psychological workings of teacher–student cultural congruence, but the broader context in which it unfolds depends largely on teachers’ ethnic identity and their approach to cultural socialization. In diverse classrooms, a teacher’s ethnic identity functions less as a demographic label and more as a pedagogical resource that carries cultural intent. The way teachers interpret and express their identities in daily interactions often shapes whether students experience a climate of acceptance, understanding, and respect. Umaña Taylor and Hill (2020) describe ethnic-racial socialization as a process through which people build cultural meaning and a sense of identity within both social and educational settings. The cultural cues teachers communicate—through language, affective support, and curriculum choices—help students understand social differences and develop stable self-concepts and emotional resilience. Wang and Huguley (2012) also showed that positive ethnic-socialization experiences protect students from the academic and emotional impact of prejudice and discrimination, highlighting the buffering role of teachers’ cultural awareness.

In this regard, teachers can act much like protective figures within families. When they approach diversity with sensitivity and empathy, students are more likely to feel safe and to sustain the motivation and emotional balance necessary for learning. Umaña Taylor et al. (2014) proposed a model of ethnic-identity development suggesting that the ways teachers express identity and position themselves culturally can offer meaning and emotional affirmation at different points in students’ identity formation, guiding their adjustment in multicultural environments. This process, however, varies across situations and is rarely linear. Benner and Graham (2013) found that discrimination from different sources affects students in distinct ways, with teachers’ implicit biases often leaving the most persistent mark. Teachers who cultivate cultural reflexivity and ethical awareness can counter such effects through empathic communication and culturally informed interpretation, helping students respond more adaptively to bias and stress. Expanding on this line of thought, Nasir et al. (2013) introduced the concept of racial storylines to describe how teachers use narrative practice and pluralistic knowledge frameworks to help students reconnect cultural identity with learning, creating new understandings of meaning and belonging. Drawing on Critical Race Theory, Pérez Huber and Solorzano (2015) examined everyday forms of cultural bias and argued that teachers who lack ethnic consciousness may unintentionally reproduce systemic inequities. By contrast, those with developed identity awareness and cultural sensitivity are more likely to recognize and address racial microaggressions, using dialogue and collaborative meaning-making to foster inclusive classroom climates.

Together, teachers’ ethnic identity and cultural socialization form the contextual ground of cultural congruence in teaching practice. Identity awareness influences how teachers feel and act in response to diversity, while socialization practices shape how students interpret culture, build trust, and develop identification. Seen this way, educational equity is less a distant institutional objective than a dynamic process created through everyday pedagogical relationships. Teachers’ self-reflection and emotional understanding are pivotal here, turning the classroom into a space where cultural understanding and psychological safety can take root. Cultural congruence, then, operates as a conditional psychological mechanism—one that influences students’ sense of security and well-being through processes such as attachment, social comparison, and intergenerational understanding. At the same time, teachers’ awareness of identity and socialization patterns alters how this mechanism appears across settings, leading to differences in its impact. Guided by these insights, the present study proposes an integrative framework to examine how teacher cultural congruence and interacting with ethnic identity and cultural socialization—shapes students’ school adaptation and mental health through the mediating role of students’ sense of belonging and emotionally supportive teacher–student interactions, thereby deepening the cultural foundations of educational equity.

## 3 Methods

### 3.1 Research design and theoretical framework

This study adopted a quantitative approach that combined survey data with structural equation modeling (SEM) to explore how teacher cultural congruence affects students’ adaptation to school through specific psychological processes. Given the cross-sectional, self-report nature of the data, all hypothesized paths were specified and interpreted as associations or predictive relations rather than causal effects. Data were obtained from three universities in western China—two ethnic universities and one teacher-training university—yielding 180 valid questionnaires. These institutions operate within a relatively standardized higher-education environment while enrolling substantial proportions of ethnic minority students, which makes perceived cultural understanding in teacher–student interaction particularly relevant for interpreting equity-related experiences.

Among the student participants, 40% identified as ethnic minorities and 60% as non-minorities. Within the teacher group, 25% were minority teachers and 75% were non-minority teachers. The gender distribution was balanced (47.8% male, 52.2% female), and the sample covered different academic years: first year (35%), second year (30%), and third year (35%). This balanced composition provided adequate representation across groups and allowed for meaningful comparison of psychological dynamics across educational levels. We acknowledge that the minority-teacher subgroup (n = 45) is relatively small for multigroup SEM; therefore, subgroup findings are interpreted cautiously as pattern-level evidence rather than definitive population estimates. The study examined four central constructs, consistent with the measurement framework specified in Table 1: teacher cultural congruence, students’ sense of belonging, teachers’ ethnic identity, and students’ school adaptation. Each construct was treated as a latent variable and measured with multiple indicators. Teacher cultural congruence consisted of three components—value congruence, cultural understanding and communication, and instructional fit. Importantly, “teacher cultural congruence” in this study refers to students’ perceived cultural congruence (i.e., perceived alignment in values, communication norms, and instructional practices) rather than objective demographic or ethnic matching. Teacher ethnicity was used only for grouping analyses to examine whether association patterns differed between minority and non-minority teachers, while the focal construct itself remained perceptual and relational in nature. Students’ sense of belonging was conceptualized as a multidimensional psychological experience encompassing emotional belonging, social belonging, and academic identification. School adaptation captured students’ functional adjustment across academic, social, and emotional domains. Teachers’ ethnic identity was assessed through indicators of ethnic self-identification and cultural integration in teaching and was modeled as a contextual moderator rather than a mediating outcome.

**Table 1:** Latent variables and observed indicators.

Latent variable	First order dimension	Sample items
A. Perceived teacher—student cultural congruence	A1. Value congruence	“My teacher respects and recognizes the values of my ethnic group.”/“My teacher and I generally share similar cultural perspectives in class.”
	A2. Cultural understanding and communication	“My teacher understands the way I express my ethnic culture and its meaning.”/“I feel that my teacher is familiar with our communication norms.”
	A3. Instructional fit	“My teacher’s instructional style feels natural and familiar to me.” / “My teacher often uses classroom examples that reflect our cultural background.”
B. Sense of belonging	B1. Emotional belonging	“I feel accepted and understood at this university.” / “I can freely express my authentic self at school.”
	B2. Social belonging	“I get along well with classmates from different ethnic groups.”/“I feel recognized and valued by members of my class.”
	B3. Academic identification	“I am proud to be a student at this university.”/“The university’s values are consistent with my own beliefs.”
C. School adaptation	C1. Academic adaptation	“I can follow the pace of class and understand the course content.” / “I am able to manage my study time and assignments effectively.”
	C2. Social adaptation	“I interact well with classmates from different ethnic backgrounds.” / “I feel comfortable and unexcluded on campus.”
	C3. Emotional adjustment	“I feel relaxed and at ease in school.”/“I rarely feel anxious about cultural differences.”
D. Teacher ethnic identity (moderator)	D1. Ethnic self identification	“I feel proud of my ethnic identity.”/“I actively express my cultural background in my teaching.”
	D2. Cultural integration in teaching	“I often incorporate ethnic cultural content into my teaching.”/“I encourage students to learn about different ethnic traditions.”
E. Gender (control variable)	E1. Gender difference	Binary variable: 0 = male, 1 = female. Female students typically report higher emotional belonging and perceived social support.
F. Grade level (control variable)	F1. Academic year distribution	Categorical variable: 1 = freshman, 2 = sophomore, 3 = junior, 4 = senior. Students in higher years tend to show more stable cultural adaptation.
G. Student ethnicity (control variable)	G1. Ethnic category	Nominal variable: 1 = minority, 0 = Han. Minority students may exhibit stronger effects along the cultural matching pathway.

The theoretical framework drew on three complementary perspectives: attachment theory, social comparison theory, and ethnic socialization theory. Attachment theory emphasizes that feelings of security are fundamental to social development; teachers’ cultural understanding and emotional responsiveness therefore help create a sense of psychological safety for students. From the standpoint of social comparison theory, students evaluate themselves in relation to teachers’ expectations and interaction styles, shaping self-evaluations and academic engagement. Ethnic socialization theory further views education as a site where cultural meanings and identities are communicated and negotiated through everyday interaction. These mechanisms are especially salient in the Chinese higher-education context, where institutional practices may be culturally standardized despite substantial student diversity, rendering classroom-level cultural recognition a key locus of students’ lived inclusion.

Grounded in these perspectives and aligned with the constructs specified in Table 1, the study hypothesized that teacher cultural congruence enhances students’ school adaptation primarily through students’ sense of belonging. Teachers’ and students’ ethnic identities were included as contextual moderators that might alter the strength of these associations. To test the model, confirmatory factor analysis (CFA) was conducted to assess the validity of each latent construct. Model fit was evaluated using CFI, TLI, RMSEA, and SRMR indices, and indirect effects were examined using bootstrap resampling procedures. To address potential common method bias arising from single-source self-report measurement, post-hoc statistical checks were conducted (reported in the Results section) to evaluate whether a single latent factor could account for the covariance among study variables. In addition, multigroup SEM analyses were performed to compare structural pathways across minority and non-minority teachers. This design ensured close alignment between theoretical assumptions, measurement structure, and empirical testing, thereby enabling a clear examination of how teacher cultural congruence operates as a psychological mechanism in shaping students’ school adaptation and experiences of educational equity.

### 3.2 Measurement framework

Drawing on validated scales commonly used in both international and Chinese research, the present study refined and contextualized the measurement instruments to fit the cultural setting of ethnic universities in China. The main variables were teacher cultural congruence, students’ sense of belonging, school adaptation, and teachers’ ethnic identity. Gender, academic year, and students’ ethnic background were included as control factors. All focal psychological constructs were measured via students’ self-reports, consistent with the conceptualization of cultural congruence as students’ perceived experience of teacher–student alignment. All items were rated on a five-point Likert scale (1 = strongly disagree, 5 = strongly agree). A pilot test confirmed acceptable reliability and validity across all measures. Teacher cultural congruence was represented by three components—value alignment, cultural understanding and communication, and instructional adaptability. These items were designed to capture perceived congruence in classroom interaction (e.g., perceived respect for ethnic-group values, perceived familiarity with communication norms, and perceived instructional fit), rather than demographic matching or objective “same-ethnicity” assignment. Students’ sense of belonging was assessed across emotional, social, and academic domains. School adaptation covered academic, social, and emotional adjustment, while teachers’ ethnic identity was measured through self-identification and cultural integration in teaching. Each observed indicator was designed to reflect both the layered characteristics of cultural psychology and the contextual dimensions of educational equity. Given that perceived congruence may be shaped by both teacher behavior and students’ interpretive frames, we interpret the construct as a relational perception that is theoretically proximal to belonging and trust formation. The complete measurement framework is shown in Table 1.

## 4 Results

### 4.1 Reliability and validity tests

Table 2 summarizes the measurement results and indicates that the latent constructs demonstrated overall acceptable and stable psychometric properties. The standardized factor loadings for teacher cultural congruence, students’ sense of belonging, school adaptation, and teachers’ ethnic identity were all statistically significant, with values ranging from moderate to high and corresponding t-values well above conventional significance thresholds (*p* < 0.001), suggesting that the observed indicators adequately captured their intended constructs. Internal consistency was supported by Cronbach’s *α* and composite reliability (CR) values that generally met or exceeded the recommended benchmark of 0.70, indicating satisfactory reliability across constructs. Evidence for convergent validity was observed for the core constructs of teacher cultural congruence and school adaptation, which exhibited comparatively stronger factor loadings and higher variance extraction, whereas teachers’ ethnic identity showed relatively lower factor loadings and variance explained, reflecting its context-sensitive and identity-related nature shaped by institutional norms and social interaction. Students’ sense of belonging and school adaptation displayed coherent loading patterns, underscoring the consistency of students’ emotional and behavioral responses. Taken together, these results support the overall adequacy of the measurement model and highlight a layered distinction between teachers’ culturally grounded practices and students’ psychological experiences of safety, identity integration, and trust. Given that all variables were collected via student self-reports, Harman’s single-factor test was conducted to assess potential common method bias; the unrotated solution did not reveal a dominant single factor, with the first factor accounting for 37.76% of the total variance, indicating that common method bias was unlikely to pose a serious threat to the validity of the observed relationships. Teacher ethnic identity showed weaker convergent validity (AVE < 0.50), which may attenuate moderation estimates; thus, moderation findings should be interpreted cautiously.

**Table 2:** Reliability and validity of constructs.

Construct	Items	standardized loading (*λ*)	t value	Cronbach’s α	CR	AVE
Teacher cultural congruence (A)	A11–A33	0.67–0.77	15.1–22.9***	0.91	0.91	0.53
Sense of belonging (B)	B11–B23	0.62–0.74	12.3–19.4***	0.85	0.85	0.48
School adaptation (C)	C11–C33	0.68–0.82	16.0–28.8***	0.92	0.92	0.55
Teacher ethnic identity (D)	D11–D23	0.46–0.61	6.1–9.0***	0.70	0.70	0.28

### 4.2 Sample characteristics and descriptive statistics

Tables 3, 4 summarize the sample characteristics and the descriptive statistics of the main study variables. The sample exhibited a balanced gender distribution, with males accounting for 47.8% and females 52.2%, and the distribution across academic years was similarly even, with first-, second-, and third-year students comprising 35.0, 30.0, and 35.0% of the sample, respectively, thereby covering key stages of university adjustment. In terms of ethnicity, 25.0% of teachers and 40.0% of students identified as ethnic minorities, reflecting an unequal representation of cultural groups within the higher-education context and implying that teacher–student cultural congruence is more likely formed through everyday interaction and negotiation in classrooms than through simple demographic matching. Descriptive analyses indicated that the mean scores for teacher cultural congruence (M = 3.05, SD = 0.82), sense of belonging (M = 3.11, SD = 0.77), school adaptation (M = 3.14, SD = 0.83), and teacher ethnic identity (M = 3.11, SD = 0.55) clustered around the mid-to-upper range of the scale, suggesting moderately positive perceived levels across constructs. Teacher cultural congruence showed the lowest mean among the four variables, while school adaptation was the highest, and the relatively close means across constructs suggest that students’ perceptions of cultural responsiveness, belonging, and adjustment were generally consistent rather than sharply differentiated.

**Table 3:** Sample characteristics.

Variable	Category	n	%
Gender	Male	86	47.8
	Female	94	52.2
Grade	Year 1	63	35
	Year 2	54	30
	Year 3	63	35
Teacher ethnicity	Minority	45	25
	Non-minority	135	75
Student ethnicity	Minority	72	40
	Non-minority	108	60

**Table 4:** Means, standard deviations, and correlations.

Variable	M	SD	1	2	3	4
1. Teacher cultural congruence	3.05	0.82	1.00			
2. Sense of belonging	3.11	0.77	0.70***	1.00		
3. School adaptation	3.14	0.83	0.66***	0.75***	1.00	
4. Teacher ethnic identity	3.11	0.55	0.04	0.02	0.15*	1.00

Correlation analyses revealed a clear and theoretically coherent pattern: teacher cultural congruence was strongly and positively associated with sense of belonging (*r* = 0.70, *p* < 0.001) and school adaptation (*r* = 0.66, *p* < 0.001), and sense of belonging was also strongly related to school adaptation (*r* = 0.75, *p* < 0.001), underscoring belonging as a central psychological connector between culturally responsive teacher–student interaction and adaptive functioning in school. By comparison, teacher ethnic identity showed near-zero correlations with teacher cultural congruence (*r* = 0.04, *p* = 0.612) and sense of belonging (*r* = 0.02, *p* = 0.823), and only a small association with school adaptation (*r* = 0.15, *p* = 0.045), indicating that teachers’ ethnic identity, as measured here, was not a primary correlate of students’ perceived cultural congruence or belonging in this sample. Overall, these descriptive and correlational results support the central role of perceived teacher cultural congruence and student belonging in shaping school adaptation and provide an empirical basis for the subsequent structural equation modeling analyses.

### 4.3 Overall effects of teacher cultural congruence on student school adaptation

Table 5 reports the baseline structural equation model without the mediator (sense of belonging), showing that teacher cultural congruence was significantly and positively associated with students’ school adaptation (*β* = 0.72, SE = 0.13, *t* = 8.04, *p* < 0.001). Given the cross-sectional design, this path is interpreted as a statistically robust association (i.e., teacher cultural congruence significantly predicted higher levels of school adaptation in the SEM) rather than evidence of a causal effect. This result indicates that when teachers bring cultural awareness, openness, and sensitivity into their teaching, students tend to report greater emotional stability and more sustained engagement in academic life; accordingly, students who perceived higher cultural congruence also reported higher adjustment. Cultural congruence may enhance the quality of classroom interactions and strengthen students’ sense of safety and trust, which can facilitate smoother engagement with school norms and peer networks.

**Table 5:** SEM main effects.

Path	Standardized β	SE	t	p
Teacher Cultural Congruence → school adaptation	0.72	0.13	8.04	< 0.001***

The findings further suggest that teachers’ cultural stance does not merely shape instructional communication—it also influences how students interpret belonging, meaning, and their place within the learning environment. Rather than reflecting a single teaching technique, cultural congruence appears to develop through teachers’ ongoing ability to recognize and respond to students’ lived experiences; when educators approach cultural differences with genuine respect and empathy, students are more likely to feel understood and accepted, which supports emotional security and day-to-day adaptation. This pattern also speaks to the broader challenge of educational equity: building cultural congruence often depends on reflective and relational work in addition to institutional policies, and teachers operating under standardized systems must continually balance consistency with culturally responsive care. Students’ responses imply that inclusion and standardization are negotiated in everyday classroom exchanges, and the extent to which schools can sustain both authenticity and equity may hinge on these routine interactions between teachers’ cultural responsiveness and students’ psychological engagement.

### 4.4 Testing the psychological mechanism: mediation of sense of belonging and school adaptation

Table 6 presents the mediation model that includes students’ sense of belonging, showing that the estimated A → C coefficient differs from the baseline model because part of the association between teacher cultural congruence and school adaptation is transmitted through the indirect pathway. The results indicate that teachers’ cultural congruence was positively and significantly associated with students’ school adaptation (direct effect *β* = 0.235, *p* < 0.001). In addition, a substantial and statistically significant indirect effect was observed through students’ sense of belonging (indirect effect *β* = 0.421, 95% CI [0.316, 0.531]), indicating that a large proportion of the association between cultural congruence and school adaptation operates via this psychological pathway. The total effect of teachers’ cultural congruence on school adaptation was also significant (*β* = 0.656, 95% CI [0.569, 0.730]), suggesting a robust overall relationship. Consistent with best practices for mediation analysis using cross-sectional SEM, these findings are interpreted as evidence of a supported indirect association rather than a temporal or causal sequence.

**Table 6:** Mediation analysis.

Path	Estimate (β)	Bootstrap SE	95% CI lower	95% CI upper
Direct effect (A → C)	0.235	0.071	0.090	0.373
Indirect effect (A → B → C)	0.421	0.055	0.316	0.531
Total effect	0.656	0.041	0.569	0.730

Substantively, higher levels of perceived teacher cultural congruence were associated with stronger feelings of belonging among students, which in turn were linked to better adaptation within the school context. Sense of belonging thus emerged as the central psychological mechanism in this process, highlighting its role in translating culturally congruent teaching practices into adaptive academic and social functioning. From a relational perspective, when students perceive their teachers as respectful of, attentive to, and responsive toward their cultural backgrounds, they are more likely to experience emotional security, recognition, and affirmation within the school environment. These experiences foster a stronger sense of belonging, which provides an affective foundation for motivation, engagement, and smoother adjustment to academic and social demands. Importantly, teachers’ cultural congruence functions not merely as an instructional characteristic but as a relational and symbolic practice embedded in everyday interactions. Through culturally attuned communication and interactional sensitivity, teachers help transform routine classroom encounters into spaces where educational equity is enacted in practice. Collectively, these findings clarify the micro-level psychological mechanism through which culturally congruent teaching supports students’ school adaptation and well-being, underscoring the central role of belonging in culturally diverse educational settings.

### 4.5 Facilitating role of minority teachers: group differences and moderating effects

**Figure 1:**
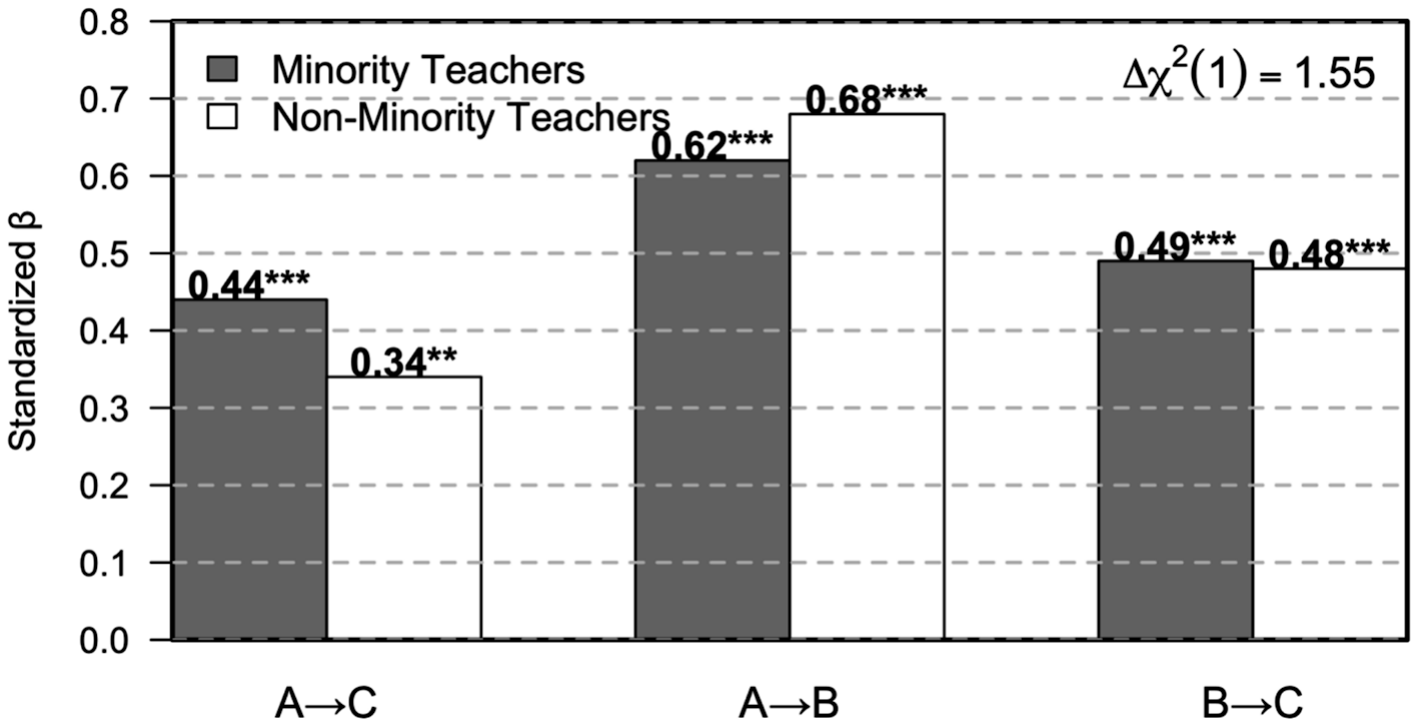
Multi group SEM path differences by teacher ethnicity.

Table 7 and Figure 1 present the results of the multi-group structural equation modeling analysis by teacher ethnicity and illustrate how teacher cultural congruence is translated into students’ adaptive outcomes under different relational contexts. Across both minority-teacher and non-minority-teacher groups, the same core structural configuration is observed, indicating that the psychological linkage among cultural congruence, sense of belonging, and school adaptation is preserved regardless of teacher ethnicity. Among minority teachers, the association between teacher cultural congruence and students’ school adaptation was relatively strong (A → C, *β* = 0.44, *p* < 0.001), alongside a substantial pathway from cultural congruence to students’ sense of belonging (A → B, *β* = 0.62, *p* < 0.001) and a robust association between belonging and school adaptation (B → C, *β* = 0.49, *p* < 0.001). This pattern suggests that, in classrooms led by minority teachers, culturally congruent teaching is closely aligned with both students’ emotional connection to the school and their broader adaptive functioning, allowing the influence of cultural understanding to be expressed in a comparatively direct and cohesive manner. In the non-minority-teacher group, all corresponding paths remained statistically significant, demonstrating that the same relational mechanism operates across groups. However, the direct association between cultural congruence and school adaptation was comparatively weaker (A → C, *β* = 0.34, *p* < 0.01), while the paths from cultural congruence to belonging (A → B, *β* = 0.68, *p* < 0.001) and from belonging to adaptation (B → C, *β* = 0.48, *p* < 0.001) remained strong.

**Table 7:** Multi group SEM by teacher ethnicity.

Group	A → C	A → B	B → C	Δχ^2^(1)
Minority teachers	0.44***	0.62***	0.49***	
Non-minority teachers	0.34**	0.68***	0.48***	1.55

The chi-square difference test indicated only modest structural variation between groups (Δχ^2^(1) = 1.55), suggesting that teacher ethnicity does not alter the fundamental psychological structure of the model, but is associated with differences in the strength and immediacy with which cultural congruence is converted into students’ adaptive outcomes. From an interpretive standpoint, this pattern implies that minority teachers’ lived cultural experiences may allow culturally congruent interaction to translate more readily into students’ feelings of legitimacy, emotional security, and engagement with school life, thereby reducing psychological distance in everyday classroom encounters. Students may experience recognition from minority teachers as more immediately grounded in shared understanding, which facilitates smoother engagement with academic norms and social relationships. At the same time, the consistently strong pathway from cultural congruence to belonging among non-minority teachers highlights that reflective cultural awareness and empathic perspective-taking can still foster students’ sense of belonging effectively, even in the absence of shared ethnic background. The comparatively weaker direct link between cultural congruence and school adaptation in this group suggests that, for non-minority teachers, the influence of culturally responsive practice may rely more heavily on students’ internalization of belonging before manifesting in broader adaptive outcomes. Viewed together, these findings position teacher identity not as a categorical determinant of effectiveness but as a contextual amplifier that shapes how efficiently relational understanding is translated into student adjustment. While minority teachers’ cultural positioning may strengthen immediacy and relational resonance, the results also point to the uneven emotional and cultural labor often placed on minority educators. Without institutional recognition and collective support for culturally responsive practice, the responsibility for fostering inclusion risks remaining concentrated at the individual level. The evidence presented in Table 7 and Figure 1 therefore underscores the importance of teacher diversity alongside sustained professional development, suggesting that cultural congruence becomes most impactful when relational sensitivity is supported as a shared pedagogical norm rather than dependent on individual identity alone.

### 4.6 Consistency of the core relational mechanism across teacher ethnicity groups

Table 8 provides a pattern-level robustness check of the core structural relations across teacher-ethnicity groups. The results show that the central relational mechanism specified in the model is broadly stable in both groups. In the non-minority-teacher group, perceived teacher–student cultural congruence (A) was strongly and positively associated with students’ sense of belonging (B) (*β* = 0.745, *p* < 0.001), and students’ sense of belonging was in turn strongly associated with school adaptation (C) (*β* = 0.761, *p* < 0.001). A highly similar pattern emerged in the minority-teacher group: cultural congruence remained a robust predictor of belonging (*β* = 0.623, *p* < 0.001), and belonging continued to show a strong positive association with school adaptation (*β* = 0.752, *p* < 0.001). Importantly, the directions of both paths are identical across groups and the magnitudes are comparably large, indicating that the psychological logic linking cultural congruence to adaptive functioning through belonging is preserved under teacher-ethnicity grouping. Although the A → B coefficient is somewhat smaller among minority teachers, the overall pattern remains coherent and consistent with the hypothesized mechanism.

**Table 8:** Pattern-level robustness of structural paths across teacher ethnicity groups.

Teacher ethnicity	A → B (β)	B → C (β)	*N*
Non-minority teachers (0)	0.745***	0.761***	135
Minority teachers (1)	0.623***	0.752***	45

At the same time, given the smaller minority-teacher subgroup (n = 45), differences in effect sizes should be interpreted cautiously as pattern-level evidence rather than definitive population estimates. Taken together, this robustness check strengthens confidence that the proposed model does not hinge on a single subgroup and that the belonging-based mechanism operates similarly across teacher-ethnicity contexts, while still leaving room for future replication with larger and more balanced samples. Whereas Table 7 examines group differences in the strength of structural paths within the full SEM framework, Table 8 serves as a pattern-level robustness check focusing on the preservation of the core A → B → C relational mechanism across teacher-ethnicity groups.

### 4.7 Evaluating the plausibility of a single-factor explanation

As shown in Table 9, the hypothesized four-factor measurement model demonstrates a substantially superior fit to the data compared with the single-factor model. The four-factor model exhibits excellent fit across all indices (CFI = 0.993, TLI = 0.993, RMSEA = 0.016), whereas the single-factor model shows notably poorer fit (CFI = 0.807, RMSEA = 0.085). This marked discrepancy indicates that the observed covariance structure cannot be adequately explained by a single latent factor. Consistent with common practice, this comparison suggests that common method variance is unlikely to fully account for the observed relationships among the study variables. Nevertheless, this analysis is treated as a diagnostic check rather than a definitive test, and the potential influence of method-related bias cannot be entirely ruled out.

**Table 9:** Comparison of single-factor and theoretical measurement models.

Model	χ^2^	df	χ^2^/df	CFI	TLI	RMSEA	N
Single-factor model	932.99	405	2.30	0.807	0.793	0.085	180
Theoretical four-factor model	417.45	399	1.05	0.993	0.993	0.016	180

### 4.8 Model comparison and robustness tests

Table 10 presents the comparison between the full mediation and partial mediation models. Both specifications exhibited excellent global fit to the data, with nearly identical fit indices (full mediation: χ^2^/df = 1.012, CFI = 0.999, TLI = 0.999, RMSEA = 0.008; partial mediation: χ^2^/df = 1.014, CFI = 0.999, TLI = 0.999, RMSEA = 0.009). The chi-square difference comparison suggested that adding the direct path did not improve model fit in any meaningful way (χ^2^diff(1) = 0.65), given the virtually identical fit indices across the two models. Accordingly, the partial mediation model does not demonstrate a clear empirical advantage over the full mediation specification. The exceptionally high fit observed for both models likely reflects the parsimonious and theory-driven nature of the specified mediation structure, as well as the use of well-aligned self-report measures collected at a single time point. Under such conditions, alternative mediation specifications that share a highly similar covariance structure are expected to produce nearly indistinguishable fit indices. From a substantive perspective, these results support the interpretation that the association between teacher cultural congruence and students’ school adaptation is primarily accounted for by indirect pathways operating through students’ sense of belonging, rather than by a stable and independent direct effect. At the same time, the near-equivalence of the full and partial mediation models suggests that, in cross-sectional data, direct and indirect relational influences may be difficult to disentangle cleanly. Differences between mediation specifications should therefore be understood as reflecting overlapping psychological processes embedded in everyday teacher–student interactions, rather than as evidence for sharply distinct causal routes. Consequently, the model comparison provides supportive—but not decisive—evidence for the proposed mediating framework, and the findings are best interpreted in terms of the internal coherence and robustness of the theoretical model rather than as confirmation that one mediation structure is definitively superior.

**Table 10:** Model comparison and robustness.

Model	χ^2^/df	CFI	TLI	RMSEA	χ^2^diff (Δdf = 1)
Full mediation	1.012	0.999	0.999	0.008	—
Partial mediation	1.014	0.999	0.999	0.009	0.650

χ^2^diff is reported as the absolute chi-square difference between nested models (Δdf = 1). The near-identical global fit indices indicate that adding the direct path does not improve model fit in a practically meaningful way.

## 5 Discussion

### 5.1 Teacher cultural congruence as a core psychological resource for school adaptation

In the baseline model without the mediator, perceived teacher cultural congruence showed a strong positive association with students’ school adaptation (*β* = 0.72, *p* < 0.001), indicating that culturally congruent teacher–student interactions are closely linked to students’ academic, social, and emotional adjustment. This effect remained substantial even before accounting for mediating mechanisms, suggesting that cultural congruence operates at a foundational level of students’ school experience. Psychologically, this pattern highlights that teaching effectiveness is not limited to the transmission of academic content but is deeply embedded in relational processes that communicate recognition, safety, and legitimacy. When teachers demonstrate sensitivity to students’ cultural backgrounds, students are more likely to interpret classroom interactions as affirming rather than evaluative, which stabilizes emotional states and supports sustained engagement with learning demands. Importantly, the strength of this association should be understood in relation to the broader institutional context. In highly standardized higher education systems, including those represented in the present sample, pedagogical norms often emphasize uniform curricula, neutral professionalism, and efficiency-oriented evaluation. Within such environments, explicit recognition of cultural difference is relatively rare. As a result, when students encounter teachers who acknowledge and respond to their lived cultural experiences, the psychological impact is amplified.

Cultural congruence becomes effective not because it is commonplace, but precisely because it disrupts an otherwise homogenizing educational climate. From a psychological perspective, this disruption restores a sense of predictability and emotional safety for students whose cultural identities may otherwise be rendered invisible or marginal. These findings also challenge the assumption that teaching can or should be culturally neutral. The strong direct association between cultural congruence and school adaptation indicates that neutrality is not experienced as neutral by students; rather, it is often interpreted as distance or indifference. By contrast, culturally congruent teaching practices signal respect and ethical recognition, allowing students to perceive themselves as legitimate members of the academic community. In practical terms, this suggests that cultural congruence functions as an equity-enabling condition: it reduces emotional friction, lowers adaptation costs, and facilitates smoother participation in academic and social life. Thus, educational equity cannot be fully realized through formal access or standardized rules alone. Instead, it emerges through everyday teacher–student interactions in which empathy, respect, and shared meaning are communicated. The present results underscore that fairness in education is not only institutional but experiential, and that teacher cultural congruence plays a decisive role in transforming equality in principle into equity as lived psychological reality.

### 5.2 Cultural congruence, teacher identity, and differential pathways to adaptation

The multi group structural equation modeling results provide important insight into how teacher cultural congruence operates differently depending on teacher ethnicity, while still following a broadly consistent psychological structure. As shown in Table 7 and Figure 1, all core pathways remained statistically significant for both minority and non minority teachers, indicating that the basic mechanism linking teacher cultural congruence, students’ sense of belonging, and school adaptation is robust across groups. However, patterned differences emerged in the strength of these associations. For minority teachers, teacher cultural congruence showed a stronger direct association with students’ school adaptation (A → C, *β* = 0.44, *p* < 0.001), alongside a strong pathway from cultural congruence to sense of belonging (A → B, *β* = 0.62, *p* < 0.001) and from belonging to adaptation (B → C, *β* = 0.49, *p* < 0.001). Among non minority teachers, although the same paths were also significant, the direct effect of cultural congruence on adaptation was weaker (A → C, *β* = 0.34, *p* < 0.01), suggesting that for these teachers the influence of cultural congruence may rely more heavily on students’ internal psychological processes before translating into broader adaptive outcomes. The chi square difference test indicated only a modest structural difference between groups (Δχ^2^(1) = 1.55), which implies that teacher ethnicity does not fundamentally alter the relational model itself but shapes how efficiently cultural understanding is converted into students’ emotional security and adjustment.

Substantively, these findings help explain why minority teachers may be particularly effective in fostering students’ adaptation through cultural congruence. Shared cultural background can provide an immediate sense of recognition and legitimacy that reduces psychological distance and strengthens students’ feelings of being understood, thereby accelerating the formation of belonging and its impact on adaptation. For students, recognition from a teacher who shares similar lived experience may feel more authentic and emotionally grounded, which can enhance trust and engagement in everyday classroom interactions. At the same time, these stronger effects should not be interpreted as a simple advantage rooted in identity alone. Minority teachers often operate under unequal institutional conditions, where cultural responsiveness is expected but not structurally supported. They are frequently positioned as informal cultural mediators who are asked to provide emotional reassurance and identity affirmation in addition to fulfilling standard professional roles. This added emotional and cultural labor helps explain why cultural congruence appears especially powerful in minority teacher contexts, but it also reveals a deeper structural imbalance. When culturally responsive practice depends primarily on individual teachers’ identity resources rather than collective norms or institutional frameworks, its benefits may be unevenly distributed and difficult to sustain.

From the perspective of educational equity, the present results suggest that teacher cultural congruence should not be viewed as a personal trait possessed by certain teachers, but as a relational capacity that can be strengthened through training, policy, and organizational support. The differential patterns observed across teacher groups highlight the need to translate the relational strengths evident among minority teachers into shared professional practices so that recognition, belonging, and adaptation do not depend on who the teacher is, but on how teaching is collectively organized. In this sense, cultural congruence emerges as both a psychological mechanism and a structural challenge, pointing to the importance of institutional responsibility in ensuring that students’ adaptation is supported through stable, equitable, and culturally responsive classroom relationships.

### 5.3 The mediation of belonging and school adaptation: how cultural congruence becomes psychologically effective

Beyond the strong overall association between teacher cultural congruence and students’ school adaptation, the mediation analysis offers a more fine-grained understanding of how this influence unfolds at the psychological level. Rather than functioning as a direct or instantaneous effect, teachers’ cultural congruence appears to operate through a gradual process in which students’ emotional experiences within the school environment are first reshaped and then translated into more stable patterns of adjustment. The results indicate that sense of belonging plays a central role in this process, serving as the key psychological mechanism through which culturally congruent teaching becomes consequential for students’ academic, social, and emotional adaptation. Specifically, when students perceive their teachers as culturally understanding, communicatively aligned, and responsive to their lived experiences, they are more likely to feel accepted, recognized, and emotionally secure within everyday classroom interactions. This sense of belonging constitutes an immediate emotional response to cultural recognition, anchoring students’ relationship with the school in feelings of safety and affirmation rather than uncertainty or distance.

Once this emotional foundation is established, students are better positioned to engage with the broader academic and social demands of university life, showing greater persistence, smoother interpersonal adjustment, and reduced psychological strain. Importantly, this pattern suggests that belonging does not merely accompany adaptation as a parallel outcome, but actively enables it by lowering the emotional costs associated with participation in school life. Students who feel that they “fit” culturally within the classroom are less likely to interpret challenges, misunderstandings, or differences as signals of exclusion, and more likely to approach academic and social situations with confidence and openness. In this sense, cultural congruence initiates a cascade of psychological changes that begin at the level of emotional experience and extend outward to shape how students navigate their educational environment. The significance of the indirect pathway, together with the stability of the total effect, supports the interpretation that cultural congruence influences school adaptation primarily by reorganizing students’ emotional orientation toward the institution before manifesting in observable adaptive outcomes. From an educational equity perspective, these findings underscore that inclusion cannot be reduced to physical access, formal representation, or policy-level guarantees of equality. Students may occupy the same classrooms and institutional spaces, yet remain psychologically marginalized if they lack a genuine sense of belonging. The present results highlight belonging as a functional psychological bridge that allows cultural recognition in teacher–student interactions to be converted into meaningful engagement and adjustment. Without such emotional grounding, structural inclusion risks remaining superficial, leaving underlying feelings of alienation intact. This helps explain why institutional reforms that focus solely on procedural fairness or demographic diversity often fail to produce durable equity outcomes: they do not necessarily address how students experience everyday interactions at the relational level. Practically, the findings suggest that efforts to support minority students’ school adaptation should prioritize the cultivation of relational climates that foster belonging rather than relying exclusively on academic support or administrative interventions. Teacher training and professional development programs that emphasize cultural awareness, empathetic communication, and sensitivity to students’ cultural backgrounds may therefore have effects that extend well beyond instructional effectiveness. By strengthening students’ sense of belonging, teachers contribute to an environment in which engagement, social integration, and emotional well-being can develop more naturally over time. In this way, cultural congruence functions not simply as an instructional characteristic, but as a relational and symbolic practice through which educational equity is enacted in everyday classroom life, transforming cultural recognition into emotional security and emotional security into sustained adaptation.

## 6 Conclusion and limitation

### 6.1 Conclusion

This study examined teacher cultural congruence and its influence on students’ school adaptation, with particular attention to the psychological mechanisms of sense of belonging, as well as the moderating role of ethnic identity. Using structural equation modeling based on 180 valid student responses, the results showed that perceived teacher cultural congruence was significantly associated with higher levels of school adaptation, both directly and indirectly. The mediation pathway through sense of belonging was statistically supported, indicating that cultural congruence operates through emotional security and relational trust to shape students’ adaptive experiences within the school context. In addition, multi-group and moderation analyses suggested that ethnic identity plays a contextual role in this process. The effects of cultural congruence on belonging and adaptation tended to be stronger in contexts involving minority teachers and among students sharing similar ethnic backgrounds, pointing to a form of cultural resonance embedded in everyday classroom interaction. Together, these findings highlight that educational equity cannot be reduced to institutional access or formal equality alone. Rather, it is also produced through teachers’ culturally attuned interactions that allow students to feel recognized, emotionally secure, and socially connected. By integrating cultural congruence, belonging, and trust into a single psychological framework, this study extends existing work on culturally responsive pedagogy and offers empirical evidence for understanding equity as a lived, relational experience within higher education.

### 6.2 Limitations and future directions

Several limitations of the present study should be acknowledged. First, although the overall sample size of 180 participants meets commonly cited minimum recommendations for structural equation modeling with a moderately complex model, it remains relatively modest for detecting more subtle effects and for ensuring highly stable parameter estimates. This constraint is particularly salient in the multi-group analyses, where the minority-teacher subgroup included only 45 participants. Such an imbalance may have reduced statistical power and increased sampling variability, suggesting that the observed group differences should be interpreted with appropriate caution. Accordingly, the multi-group results are best understood as pattern-level evidence rather than definitive population-level estimates. While the direction, magnitude, and internal coherence of the estimated paths were theoretically meaningful and empirically interpretable, future studies should seek to replicate these analyses using larger and more balanced samples across teacher ethnic groups. Employing multi-site or longitudinal designs would further strengthen the robustness of these findings and help clarify whether the moderating role of cultural congruence remains stable across institutional contexts and over time.

In addition, intraclass correlations were not estimated because the available school-level clustering information was insufficient to support meaningful variance partitioning. Specifically, the number of participating schools was small and the distribution of cases across schools was highly unbalanced, rendering ICC estimation statistically inappropriate in the present dataset. This design constraint limits the extent to which between-school variance can be evaluated and should be taken into account when interpreting the results. A second limitation concerns the cross-sectional nature of the data and the exclusive reliance on student self-report measures. Although post-hoc statistical checks suggested that common method bias was unlikely to fully account for the observed associations, the study design does not permit causal inference. Longitudinal approaches would allow future research to examine how teachers’ cultural congruence and students’ sense of belonging dynamically develop and jointly shape students’ longer-term academic and social adaptation. Moreover, incorporating multiple data sources—such as teacher self-reports, peer evaluations, or classroom observations—could provide a more comprehensive understanding of how cultural congruence is enacted in everyday instructional practice. Despite these limitations, the present study offers a theoretically grounded and empirically supported framework for understanding the psychological foundations of educational equity. By situating its conclusions within clearly articulated methodological boundaries, it provides a transparent and credible basis for future research aimed at deepening and extending investigations of cultural congruence and student adaptation in culturally diverse educational settings.

## Data Availability

The raw data supporting the conclusions of this article will be made available by the authors, without undue reservation.
